# Evaluation of redevelopment priority of abandoned industrial and mining land based on heavy metal pollution

**DOI:** 10.1371/journal.pone.0255509

**Published:** 2021-07-29

**Authors:** Xing Gao, Junping Tian, Zheng Huo, Yanbin Wu, Chenxi Li

**Affiliations:** 1 School of Public Administration, Hebei University of Economics and Business, Shijiazhuang, China; 2 Hebei Province Geographic Information Platform for Socio-Economic and Social Development, Hebei University of Economics and Business, Shijiazhuang, 050061, China; 3 School of Information Technology, Hebei University of Economics and Business, Shijiazhuang, China; 4 School of Public Administration, Xi’an University of Architecture and Technology, Xi’an, China; Northeastern University (Shenyang China), CHINA

## Abstract

Heavy metal contamination in soil is an important factor affecting the determination of safe redevelopment methods for industrial and mining land. In this paper, the soil environment of a typical mining city in northern China was taken as the research object, 148 surface soil samples were collected and the contents of heavy metals were measured. The health risk classification criteria for heavy metal contamination of soils and the method of priority assessment for redevelopment were used. The results showed that: the risk of potential utilization types of heavy metals in the abandoned industrial and mining land is different. When the utilization type is agricultural land, the soil environmental quality is good as a whole, and a small number of plots are polluted by cadmium (Cd)and mercury (Hg); When the land use type is construction land, the risk of heavy metal pollution comes from chromium (Cr); The priority of development in this study area is as follows: agricultural land > construction land > ecological land.

## 1 Introduction

Abandoned industrial and mining land, as well as construction land of transportation infrastructure and water conservancy, is prominent in resource-based cities [1,2]. These land uses waste a large amount of substantial land resources and forms have significant soil pollution, especially because of heavy metals [3,4]. In recent years, with the continuous progress of global urbanization, the adjustment of industrial structure and layout has been accelerated, the transformation and redevelopment rate of abandoned industrial and mining land has been accelerated, and a large number of them has been converted into residential, school or food crop production and other sensitive land types, the potential health risks are also different [5–10]. Compared with developed countries, developing countries are facing the shortage of funds and technologies to control soil heavy metal pollution, especially in Asia and Africa [11]. In order to achieve the goal of Zero Hunger (Goal 2), Sustainable Cities and Communities (Goal 11) proposed by the Sustainable Development Goals (SDGs), these countries must ensure the safe, low-cost and efficient redevelopment of abandoned industrial and mining land in resource-based cities [12–14]. Therefore, in order to ensure the safety of land redevelopment and the sustainable and healthy development of the city, it is urgent to deeply explore the pollution characteristics and risk level of typical soil heavy metals in the process of urbanization, in order to provide reference for the scientific management and effective treatment of abandoned industrial and mining land in urban redevelopment and utilization [15–17].

Researchers have performed various studies on the evaluation of heavy metal soil pollution and redevelopment of abandoned industrial and mining sites; however, most of these studies only focus on evaluating methods for a single pollution area or redevelopment type, overlooking their interdependency. Among them, the main evaluation methods for regional heavy metal soil pollution include, Nemero comprehensive pollution index, geo-accumulation index, and potential ecological hazard index [18–23]. These methods have been widely applied for the risk evaluation of heavy metal soil pollution, however, there are some restricts, for example, Potential ecological risk assessment method is difficult to directly reflect the impact of heavy metals on human health, Nemero comprehensive pollution index and geo-accumulation index are not suitable for the assessment of health risk in abandoned industrial and mining land [24–28]. Therefore, it is important to develop a novel method to evaluate the risk of the abandoned industrial and mining land during redevelopment. Moreover, it can be used as a reference for redevelopment priority. According to China’s Ministry of Ecology and Environment released the Soil Environmental Quality Soil Contamination Risk Control Standards for Agricultural Land (for Trial Implementation, GB15618-2018) and Soil Environmental Quality Soil Contamination Risk Control Standards for Construction Land (for Trial Implementation, GB36600-2018) in August 2018. Six kinds of heavy metals, such as arsenic (As), cadmium (Cd), chromium (Cr), copper (Cu), lead (Pb), and mercury (Hg), are listed for risk evaluation, however, these two Standards only purposed the environmental risk of single heavy metal, lacking the integrated pollution risks of total six heavy metals. Abandoned industrial and mining land has great potential for redevelopment, and the types of redevelopments mainly correspond with different priorities such as agricultural land, industrial site tourism, ecological land, and construction land [29–41]. However, how to determine the priority of different types of redevelopment land needs further study. It should be high-lighted that originally the abandoned industrial and mining land is affected by the different degrees of heavy metal contamination in soil. Whereas, formerly the redevelopment types proposed under the influence of technical standards, industry standards, or policy restrictions, coupled with the existence of artificially set priorities of agricultural land, ecological land, construction land, and other fixed single types of priority, the redevelopment types for abandoned industrial and mining land in the landscape spatial structure is not considered enough [42,43]. The spatial distribution of heavy metal contaminated soil has been neglected to influence the land use functions embedded in different types of redevelopments of abandoned land.

Thus, in this paper, a novel evaluation method is purposed using the integrated pollution risks of total six heavy metals (As, Cd, Cr, Cu, Pb, and Hg), which is further employed to evaluate redevelopment priority of abandoned industrial and mining land. Taking a resource-based city in Hebei Province as the research area, 148 pieces of abandoned industrial and mining land for redevelopment were selected. According to different potential utilization types of land, the risk screening value and risk control value were integrated, and the health risk classification standard of soil heavy metal pollution was constructed. Furthermore, on the basis of the spatial connectivity of landscape, a method for measuring the redevelopment priority of industrial and mining waste land under different utilization scenarios is proposed. This method is used to measure the priority of 148 abandoned industrial and mining land. It can be used to provide reference for scientific management and effective governance of abandoned industrial and mining land for urban redevelopment.

## 2 Study area and data sources

### 2.1 Study area

The study area is located in the south of Hebei Province, at the eastern foot of the Taihang Mountains, at the junction of Shanxi, Hebei, and Henan provinces. This area has a population of about 530000 with a total area of 353 km^2^, including agricultural, construction, and other land areas of 15831.57 hm^2^, 8324.11 hm^2^, and 11144.32 hm^2^, respectively. As a typical resource-based old industrial mining area, the mineral resources in the study area are widely distributed, with rich reserves of coal, porcelain clay, limestone, and other minerals. The traditional industries, such as the coal chemical industry, iron and steel, ceramics, and building materials that accompany the development of mineral resources, cover a large area. Because of the depletion of mineral resources and the transformation and upgrading of industries in recent years, many industrial and mining sites have been abandoned. Field surveys have shown that, the soil environment has suffered different degrees of impact and damage because of mineral development and industrial production. A total of 148 land plots have been destroyed and suppressed, with a total area of 671.87 hm^2^.

### 2.2 Data sources

There are 148 abandoned industrial and mining sites in this study area, and one sampling site was created for each site, resulting in a total of 148 sampling sites. Each sample consisted of 2–4 subsamples, each subsample was mixed thoroughly, and 1000 g of each was packed in a polyethylene self-sealing bag. The soil samples were air dried in a cool place, debris was removed, and the samples were then ground, and passed through 100 mesh sieves. Soil pH was analyzed by potentiometry, using an acidity meter, glass electrode, and pH compound electrode. Hg and As were determined using an AFS-3100 atomic fluorescence spectrometer (Beijing Haiguang Instruments Co., Ltd., Beijing, China). Cd, Cu, Pb, and Cr were determined by atomic absorption spectrometry (Pin Add 900T, PerkinElmer, USA). All measurements were conducted three times to confirm the accuracy.

## 3 Methodology

### 3.1 Methodology for evaluating the risk of heavy metal contamination

The evaluation criteria for the exceedance of heavy metal pollution standards of soil in this study area were based on the Soil Environmental Quality Risk Control Standards for Soil Contamination on Agricultural Land (Trial; GB15618-2018) and the Soil Environmental Quality Risk Control Standards for Soil Contamination on Construction Land (Trial; GB36600-2018). The arable land type in this study consisted of dry land, and the soil pH values were all > 7.5. The evaluation criteria for exceeding the heavy metal standards in soil are shown in Table 1. The two standards mentioned above stipulate that agricultural land is used as defined in the GB/T21010 standard, which specifically includes arable land, garden land, and grassland. The first type of construction land includes residential land in urban construction land as stipulated in GB50137, public administration and service land for primary and secondary schools, medical land, social welfare facilities, and community and children’s parks in parkland. The second type of construction land includes industrial land, logistics and storage land, commercial service facilities, roads and transportation facilities, public facilities, public administration and service land in urban construction land under GB50137, green areas and squares (except for community or children’s park sites in G1).

**Table 1:** Evaluation criteria for excessive heavy metal contamination of soil in the study area.

Heavy metal types	Soil contamination risk of agricultural land		Soil contamination risk of construction sites			
	Screening values	Intervention values	Type I sites		Type II sites	
			Screening values	Intervention values	Screening values	Intervention values
As	25	100	20	120	60	140
Cd	0.6	4	20	47	65	172
Cr	250	1300	3	30	5.7	78
Cu	100	-	2000	8000	18000	36000
Pb	170	1000	400	800	800	2500
Hg	3.4	6	8	33	38	82

Unit: mg/kg.

The two standards, (GB15618-2018) and (GB36600-2018) specify the risk screening and intervention values for soil contamination on agricultural and construction land, respectively. If the content of contaminants in the soil is equal to or lower than the risk screening value, the health risk is negligible. The risk screening value refers to the general sense that the health risk is negligible if the content of contaminants in the soil is equal to or lower than this value. Risk intervention values refer to soil contaminant levels above which there is a generally unacceptable risk to human health. The development of screening and regulatory value thresholds in the standard is based on the risk to human health through the migration of contaminants in soil to humans. Typically, ecological sites are less likely to produce products for consumption and people are less likely to come into direct contact with the soil in the area; thus, its risk is generally not evaluated. Therefore, in this study, control measures based on screening and intervention values were developed for the selection of safe land use types for abandoned industrial and mining sites. Additionally, a grading criterion was developed to evaluate the health risk of individual and combined heavy metal contamination in soil for agricultural and construction land (Table 2).

**Table 2:** Health risk classification criteria for heavy metal contamination of soils.

risk level	Risk of individual heavy metal contamination (X)	Integrated pollution risks	Characterization of the contamination risk levels of contamination for agricultural land	Characterization of contamination risk levels for construction sites
I	X ≤ risk screening value	(Y1,Y2. . .Y6)max = Ⅰ	Low and generally negligible risk of soil contamination on agricultural land	Negligible risk to human health from soil contaminants in construction sites
II	Risk screening value < X ≤ risk intervention values	(Y1,Y2. . .Y6)max = Ⅱ	Possible risk of soil contamination such as non-compliance with quality and safety standards for edible agricultural products	Possible risks to human health from soil contaminants in construction sites
III	X> Risk intervention values	(Y1,Y2. . .Y6)max = Ⅲ	High risk of soil contamination on agricultural land such as food produce not meeting quality and safety standards	Soil contaminants in construction sites present an unacceptable risk to human health and risk management or remediation measures should be taken

### 3.2 Scenario design for potential types of redevelopments of abandoned industrial and mining sites

According to the policy requirements and years of practice, the three potential redevelopments types of abandoned industrial and mining land are agricultural land, construction land, and ecological land. The scenario design selection was based on the assessment of soil contamination risk status, and the type of redevelopment of abandoned industrial and mining land was gradually selected according to the comprehensive risk level of land contamination. Patches with level III comprehensive risk were not considered to be suitable for redevelopment until they can be rehabilitated to a safe use level according to the control measures. Additionally, as the land use map cannot distinguish between the two types of construction land, they were combined and the stricter standards were used to evaluate the pollution risk level.

1. Priority scenario for agricultural land (usually cropland and garden land). The reclamation of abandoned industrial and mining land as agricultural land has interminably been the priority, due to the practical needs of agricultural land protection. Abandoned industrial and mining land that meets the level I and II comprehensive pollution risk requirements of agricultural land can be redeveloped as agricultural land, while the lands that meet the level III comprehensive pollution risk requirements of agricultural land and levels I and II comprehensive pollution risk requirements of construction land can be redevelopment as construction land. The lands that do not meet either of the two, were set as forest land.
2. Priority scenario for construction land. Abandoned industrial and mining land belongs to construction land in the current land use classification, and direct utilization conversion cannot consider new construction land indices to reduce the pressure on the balance of occupation. The abandoned industrial and mining land that meets the level Ⅰ and Ⅱ comprehensive pollution risk requirements of construction land can be redeveloped as construction land, while lands that meet the level Ⅰ and Ⅱ comprehensive pollution risk requirements of agricultural land can be redeveloped as arable land. Those that do not meet these requirements need to be set as forest land.
3. Priority scenario for ecological land (usually forest land and grassland). The restoration to woodland and grassland requires less investment and is easier, so it can be used as a reserve in case of future requirements of arable and construction land, and to enhance the regional land ecology. Therefore, the abandoned industrial and mining land was set to be entirely redeveloped as forest land.

### 3.3 Prioritization of redevelopment of abandoned industrial and mining sites

#### 3.3.1 Establishing a priority evaluation indicator system

The multi-solution and multi-objective nature of abandoned industrial and mining land redevelopment has been verified in several studies. The challenge of determining the final type of redevelopment, has undergone several stages of theoretical guidance from academics and practitioners: from the priorities of agricultural land that simply pursues increased production, construction land that pursues economic use value, or ecological land that pursues higher ecological service value, to the maximization of the combined benefits of nature, economy, and ecology [44,45]. The index system constructed in this study was first assessed by each single index, then integrated and calculated to determine the priority. Finally, the priority was used to measure the redevelopment priority of the types of abandoned industrial and mining land under different potential scenarios. This approach was adopted based on the following three considerations.

1. The redevelopment of abandoned industrial and mining land is a typical landscape change for land that was severely disturbed by human beings, and the description of this process should grasp the organic unity of its structural and functional changes. The constructed index system should reveal the natural properties and spatial characteristics of the abandoned industrial and mining sites, and portray the ecological processes and functions associated with them Additionally, the constructed index system should reflect the changes and inner mechanisms in biodiversity, land use restructuring, corridors (such as roads and water systems), urban planning, etc.
2. As a special type of land use, the redevelopment process of abandoned industrial and mining land is a dynamic process that integrates ecological, economic, and social attributes. This process is subjected to the joint action of many factors such as pollution status, topography, patch size, spatial distribution, surrounding landscape, and economic development level. Additionally, the dynamic mechanism and evolution process of the redevelopment process are different. Regardless of the final redevelopment type, judging the degree of its merit should follow the fundamental guidelines of promoting productivity, improving the quality of the ecological environment, enhancing the function of the ecosystem, and ensuring integration and unity with the surrounding ecological landscape system at a certain spatial scale.
3. Starting from the core idea of the system theory that “structure determines function,” the redevelopment of abandoned industrial and mining land as a system requires the definition of an index that can reflect the degree of interactions among various factors and their attributes. Simultaneously, combined with the practical considerations of planning and design for the redevelopment of abandoned industrial and mining land, the “priority” index was defined to reflect the point-in-time state of the abandoned industrial and mining land redevelopment system. This system was used to characterize the division of labor and collaboration among the elements, and achieve the optimal comprehensive function.

#### 3.3.2 Prioritization assessment indicator system and content interpretation

Based on the above analysis, the specific process was as follows: using the landscape ecology method and Fragstats 4.2 software, the number of patches (NP), maximum patch area index (LPI), shape index (SHAPE), aggregation index (CONTAG), proximity index (PROX), Shannon’‘s diversity index (SHDI), and an index system of eight indices, including the structural connectivity index (COHESION) and functional connectivity index (CONNECT) were selected, following the principles of systematicity, dominance, variability, and measurability, to build a priority assessment index system for the redevelopment of abandoned industrial and mining sites in the study area. The above mentioned eight indices were integrated to calculate the priority through hierarchical analysis.

In addition to their own landscape ecology definitions, these eight indices somewhat reflect the influence of human activities in the study area in terms of natural conditions, social cognitive level, and economic development level. Among them, the NP and LPI somewhat reflect the degree of artificial intervention in the landscape change process, while the degree of human activity intervention can reflect the level of economic development of the study area. The SHAPE and SHDI can reflect the natural conditions of the plot before the cessation of industrial and mining production activities. CONTAG, PROX, COHESION, and CONNECT can reflect the degree of spatial concentration and contiguity with the surrounding patches and influence the determination of the redevelopment mode in terms of engineering economy, utilization convenience of the surrounding facilities, accessibility, and social carrying capacity. Biodiversity often serves as a reference for the subsequent landscape and urban planning schemes of the patches (Table 3).

**Table 3:** Description of landscape index.

Subgoal layer	Normative level	Formula description	Index connotation
richness	NP	*NP* = *N* Where *N* is the total number of patches in the landscape.	NP takes a value of NP ≥ 1. When to the value = 1, there is only one patch of that patch type in the entire landscape.
	LPI	$LPI=\frac{\max_{j=1}^{a}(a_{ij})}{A}$ where a_*ij*_ is the area of the patch *ij*; A is the total area of the landscape including the internal background.	LPI is the area of the largest patch in each patch type divided by the area of the entire landscape and then converted into a percentage. The range of values is 0 < LPI ≤ PI, and as it approaches zero, the area of the largest patch in this patch type becomes smaller; when it equals 100%, the entire landscape consists of a single patch.
dominance	SHAPE	$SHAPE=\frac{0.25p_{ij}}{\sqrt{a_{ij}}}$ where a_*ij*_ is the area of patch *ij*, and *p*_*ij*_ is the perimeter of patch ij.	SHAPE eliminates the effect of changes in the perimeter area ratio due to changes in plaque area in the perimeter area ratio by cross-referencing to a square standard.
	SHDI	$SHDI=-\sum_{i=1}^{m}\left(p_{i}\times \ln p_{i}\right)$ where *p*_*i*_ is the area share of patch *i* in the landscape.	The value of this indicator SHDI ≥ 0. SHDI = 0 when there is only one patch in the entire landscape, and the value increases as the number of patch types in the landscape increases and their area weight equalizes.
coherence	CONTAG	(100) $CONTAG=[1+\frac{\sum_{i=1}^{m}\sum_{j}^{m}\left[\left(p_{i})[\frac{g_{j}}{\sum_{j=1}^{m}g_{ij}}\right]\right]•[\left[\ln(p_{i})][\frac{g_{j}}{\sum_{j=1}^{m}g_{ij}}\right]]}{2\ln(m)}]100$ where *p*_*i*_ is the proportion of the total landscape area occupied by the ith landscape type, m represents the number of landscape types, and g_*ij*_ is the probability that two randomly selected adjacent rasters belong to types *i* and *j*	CONTAG is often used as a measure of the complexity of the landscape patch shape. The range of values is 0 < CONTAG ≤ 100. Generally, small values of CONTAG indicate the presence of many small patches in the landscape; convergence to 100 indicates the presence of dominant patch types with extremely high connectivity in the landscape.
	PROX	$PROX=\sum_{i}^{n}\frac{a_{ijs}}{h_{ijs}^{2}}$ where *a*_*ijs*_ is the area of patch *ijs* within the search radius from patch *ij* (m^2^), and *h*_*ijs*_ is the distance from patch ij to patch *ijs*, specifically the distance from the center of the patch edge raster to the center of the central patch edge raster.	PROX is used to describe the proximity between patches of the same type. It is a dimensionless unit and where PROX ≥ 0. If a patch has no patches of the same type as it is within the search radius, its value is zero. The value of PROX increases as the number of patches of the same type in the immediate area increases, and as the proximity and compactness of these patches increases.
	COHESION	$COHESION=[1-\frac{\sum_{j=1}^{n}p_{ij}^{*}}{\sum_{j=1}^{n}p_{ij}^{*}\sqrt{a_{ij}^{*}}}]÷[1-\frac{1}{\sqrt{Z}}]\times 100\%$ where *p*_*ij*_ is the jth perimeter of the ith class patch, *a*_*ij*_ is the jth area of the ith class patch, Z is the total number of rasters in the landscape, and n is the total number of patches of the jth class	The structural connectivity index is used to describe the physical connectivity of the landscape type. Higher COHESION values reflect better landscape connectivity with values in the range of 0–100%
	CONNECT	$CONNECT=\frac{\sum_{j\neq k}^{n}C_{ijk}}{\frac{n_{i}(n_{i}-1)}{2}}\times 100$ where *C*_*ijk*_ indicated connection between patches j and k (0 = unjoined, 1 = joined) of the corresponding patch type (*i*), based on a user specified threshold distance, and *n*_*i*_ is the number of patches in the landscape of the corresponding patch type (class)	Functional connectivity refers to the continuity of the landscape as reflected by the ecological processes and functional relationships of the landscape elements as the main characteristics and indicators. The higher the CONNECT value, the better the response to the degree of landscape connectivity, with values between 0 and 1.

#### 3.3.3 Priority indicator weights

Hierarchical analysis was used to determine the weights of priority indicators [46,47]. First, a hierarchical analysis structural model was established, and the indicator system was divided into three dimensions: richness, dominance, and contiguity, where each dimension contained several indicators. Domain experts assigned values according to the importance of the indicators and constructed a judgment matrix for each layer. The matrix was tested for consistency and if it passed the test, the eigenvector, corresponding to the largest eigenvalue, was noted as the weight. The consistency index was calculated as CI = (λmax-n)/(n-1), where n denotes the order of the judgment matrix. To measure the size of CI, the random consistency index, RI = [0, 0.58, 0.90, 1.12, 1.24, 1.32, 1.41, 1.45, 1.49, 1.51], and consistency ratio CR = CI/RI, were introduced, with the matrix considered to pass the consistency test at CR < 0.1. The final weights of NP, LPI, SHAPE, CONTAG, PROX, SHDI, COHESION, and CONNECT were 9.23, 13.15, 15.54, 17.23, 12.11, 9.09, 11.72, and 14.16, respectively.

#### 3.3.4 Prioritization evaluation model

After determining the weights of the priority degree indicators, the weighted sum of the indicators was calculated to obtain the priority of the redevelopment of abandoned industrial and mining sites in the study area. The importance of the priority degree was set using different indicators to assign weights to priority of the study area, i.e., the indicators that are conducive to reducing landscape fragmentation are assigned higher corresponding weights. Therefore, the larger the indicators weighting value, the higher the spatial connectivity of the regional landscape, with the following formula:

$$F=\sum_{i=1}^{n}W_{i}R_{i}$$ (1)

where F is the priority, *W*_*i*_ is the weight of the *i* th landscape indicator, *R*_*i*_ is the standard value of the corresponding landscape index of the *i* th landscape indicator, calculated according to the formulas in Table 3, and n is the number of indicators, which is eight in this case.

Since the range of values taken by each landscape index varies widely, the mean was calculated to de-quantize the landscape index.


$$R_{i}=\frac{x_{i}}{\bar{x}}$$ (2)

Of which

$$\bar{x}=\frac{1}{n}\sum_{i=1}^{n}x_{i}$$ (3)

where *R*_*i*_ is the standard value of the *i* th landscape index, *X*_*i*_ is the value of the *i* th landscape index, $\bar{X}$ represents the average value of the landscape index, and *n* is the priority scenario, which is three in this case.

## 4. Results

### 4.1 Statistical characteristics of heavy metals in soils

The results of descriptive statistics of heavy metal content in soil of the study area (Table 4) showed that the average contents of Cu, Pb, and Hg were 49.82, 48.62 and 0.75 mg/kg, respectively, which were 1.82, 2.37, and 30.51 times, higher than the background values of the soils in Hebei Province. Additionally, the multiplier of Hg was relatively larger, indicating that Hg accumulated in the soil to some extent. Compared with the criteria for evaluating the excess heavy metal pollution in soils of the study area in Table 1, the average contents of the six heavy metals did not exceed the screening and intervention values for heavy metal pollution in soils of agricultural and construction land. The coefficients of variation of heavy metals were Cr>Hg>Cd>Pb>Cu>As, among which the coefficients of variation of Cr, Hg, and Cd were 100%, demonstrating strong variability, indicating that their spatial variability was high and influenced by human factors. The remaining three heavy metals showed moderate variability, and their spatial variability was relatively low.

**Table 4:** Descriptive statistics of heavy metal content in soil of the study area (mg/kg).

Item	As	Cd	Cr	Cu	Pb	Hg
Minimal value	3.10	0.10	0.80	17.89	3.91	0.05
Maximal value	15.50	3.07	108.20	88.56	102.00	4.59
Median	7.80	0.26	2.50	56.00	54.36	0.16
Average value	8.13	0.29	4.70	49.82	48.62	0.75
Background value	12.60	0.56	65.40	27.30	20.50	0.02
(statistics) standard deviation	2.74	0.31	13.86	20.19	24.54	0.85
Coefficient of variation (%)	33.69	107.10	294.72	40.53	50.47	112.47

### 4.2 Spatial distribution characteristics of heavy metal pollution risk in soil

#### 4.2.1 Risk of single heavy metal contamination on agricultural land

All plots in this study were at risk of level I heavy metal pollution by As, Cr, Cu, and Pb. One plot in the study area had a Cd pollution risk of level III, covering an area of 22.63 hm^2^, which accounts for 3.37% of the total area of abandoned industrial and mining land. There were 10 plots with a Cd pollution risk of level II, covering a total area of 36.44 hm^2^, which accountings for 5.42% of the total area of abandoned industrial and mining land. The remaining plots had a Cd pollution risk of level I. In the study area, two plots had a Hg pollution risk of level III, covering a total area of 2.68 hm^2^, which accounts for 0.40% of the total area of abandoned industrial and mining land. Eight plots had a Hg pollution risk of level II, and a total area of 33.76 hm^2^, accounting for 5.02% of the total area of abandoned industrial and mining land. The remaining plots had a Hg pollution risk of level I.

#### 4.2.2 Single heavy metal contamination risk of type I sites on construction land

All plots in this study had a level I risk of heavy metal pollution by As, Cd, Cu, Pb and Hg. The risk of Cr contamination was level III in 38 plots in the study area, covering a total area of 382.123 hm^2^, and accountings 56.87% of the total area of abandoned industrial and mining land, distributed in every town, except H. The risk of Cr contamination in the remaining plots was level I.

#### 4.2.3 Single heavy metal contamination risk on type II sites on construction land

All the plots in this study had a level I risk of heavy metal pollution by As, Cd, Cu, Pb, and Hg. There were 17 plots with a level Cr III pollution risk, covering a total area of 115.08 hm^2^, accountings for 17.13% of the total area of abandoned industrial and mining land. There were 21 plots with a level II pollution risk of Cr, covering a total area of 267.04 hm^2^, accountings for 39.75% of the total area of abandoned industrial and mining land. The remaining plots had a level I pollution risk for Cr.

#### 4.2.4 Comprehensive pollution risks

In the study area, three agricultural lands had a comprehensive pollution risk of level III, covering a total area of 25.31 hm^2^, accountings for 3.76% of the total area of abandoned industrial and mining land. There were eight plots of agricultural land with a comprehensive pollution risk of level II, covering a total area of 33.76 hm^2^, which accounts for 5.02% of the total area of abandoned industrial and mining land. The remaining agricultural land had a comprehensive pollution risk of level I. There were 38 plots of type I construction land with a level III comprehensive pollution risk, covering a total area of 382.12 hm^2^, accountings for 56.87% of the total area of abandoned industrial and mining land. The remaining type I construction lands had a level I comprehensive pollution risk. There were 17 type II construction sites with a comprehensive pollution risk of level III, covering a total area of 115.08 hm^2^, accountings for 17.13% of the total area of abandoned industrial and mining land. There were 21 type II construction sites with a comprehensive pollution risk of level II, covering a total area of 267.04 hm^2^, which accounts for 39.45% of the total area of abandoned industrial and mining land. The remaining type II construction lands had a comprehensive contamination risk of level I.

### 4.3 Priority analysis of the types of safe redevelopment

#### 4.3.1 Fragstats model spatial scale

Based on the raster data requirements of the Fragstats model and the raster size simulation experiments, this study used a starting raster size of 100 × 100 m, and gradually increased the spatial distribution rate in 10 m units. All land use data were transformed into 30 × 30 m raster data in the same projection coordinate system to calculate the values of each landscape index. According to the current land use situation, the land use types in this study area were simplified into five categories: agricultural land, construction land, forest land, unused land, and water area.

#### 4.3.2 Evaluating the redevelopment priority of abandoned industrial and mining sites

The current land use map, and the land use map under different redevelopment scenarios, were recorded into the Fragstats model to calculate the following eight indices, NP, LPI, SHAPE, CONTAG, PROX, SHDI, COHESION, and CONNECT. The total area of abandoned industrial and mining land in this study was only 0.19%, which is relatively small, considering that if the spatial structure analysis of the entire area is conducted, the scale will be too large, resulting in minor differences in the values and difficulties in differentiating priority. Therefore, this study used a starting point of 100 m, and gradually increased the analysis range by 100 m until reaching 1000 m. Results show that differentiation was optimal at 500 m. Therefore, the area of 9864.27 hm^2^ in the 500 m range around the abandoned industrial and mining land was determined as the analysis range, consequently, accounting for 6.8% of the entire area.

As shown in Table 5, the F-values of the three priority scenarios for agricultural, construction, and ecological land are 115.93, 96.13, and 93.07, respectively (Table 5), with the highest priority for the agricultural land scenario and the lowest priority for the ecological land scenario. Thus, from the perspective of prioritization, the redevelopment of abandoned industrial and mining land should be concentrate on agricultural land under the premise of safe soil use.

**Table 5:** Evaluation of spatially concentrated contiguity.

Redevelopment scenarios	NP	LPI	SHAPE	PROX	CONTAG	SHDI	COHESION	CONNCT	*F*
Priority for agricultural land	1342	5.85	4.12	325.69	48.29	1.18	95.81	0.48	115.93
Priority for construction land	1338	5.04	3.43	281.49	45.68	1.15	92.40	0.32	96.13
Priority for Ecological land	1339	4.89	3.33	253.35	42.33	1.13	95.81	0.32	93.07

Compared the research results with the urban planning of the study area. Goals based on food security, the main redevelopment direction of abandoned industrial and mining land is agricultural land in study area, and other plots plan to carry out remediation of soil pollution. The results are very consistent with the reality. Therefore, the research method has high feasibility.

## 5. Discussion

Based on the results of heavy metal health risk assessment of abandoned industrial and mining land, this study constructed the evaluation method system of redevelopment priority, and analyzed the priority of redevelopment land use types from the perspective of landscape spatial connectivity. Compared with other methods, the risk assessment method of soil heavy metal pollution in this study has the advantages of convenience and clear basis for risk classification [48]. The evaluation results are consistent with the actual urban development planning of the study area, which further shows that the evaluation index and model results are reasonable. The research results can effectively measure the spatial distribution characteristics of heavy metal pollution in abandoned industrial and mining land of resource-based cities, and provide reference for the sustainable use of urban land. However, this study did not evaluate the suitability of abandoned industrial and mining land reclamation for agricultural land. In the future, priority can be studied based on the suitability evaluation of soil texture, reclamation difficulty, soil fertility, soil and water and other conditions [49–52]. At the same time, this study only analyzed the risk status of soil pollution, and did not consider the impact of soil heavy metal remediation on redevelopment. In the aspect of priority evaluation, although we try to build an evaluation system from the aspect of landscape spatial connectivity, because the area of abandoned industrial and mining land in the study area accounts for a small proportion of the whole area, the 500m area around the abandoned industrial and mining land plot is used as the calculation scale, and the impact of different spatial scales on the results still needs to be further tested. To explore its influence on the calculation results.

## 6. Conclusions

This study developed a set of assessment methods for the redevelopment of abandoned industrial and mining sites on a macroscopic scale based on two dimensions of heavy metal soil contamination risk assessment and redevelopment priorities. Further, it provided a reference for the redevelopment of abandoned industrial and mining sites with soil contamination. The results for heavy metal soil pollution risk evaluation show that the single heavy metal pollution risks for agricultural land are mainly posed by Cd and Hg, and 3.76% and 5.02% of abandoned industrial and mining land belong to the level III and II pollution risk, respectively. Single heavy metal pollution in construction land occurs because of Cr, and 56.87% of abandoned industrial and mining land is type I construction land with level III pollution risk. However, 17.13% and 39.75% of abandoned industrial and mining land are type II construction land with level III and II pollution risk, respectively. In general, the risks of potential utilization types of heavy metals in the abandoned industrial and mining land are different. When the utilization type is agricultural land, the soil environmental quality is good as a whole, and a small number of plots are polluted by Cd and Hg; When the land use type is construction land, the risk of heavy metal pollution comes from Cr. Cd, Hg and Cr in the soil are obviously enriched, which may be related to mining and industrial processing in the study area. The results for the redevelopment priority degree of abandoned industrial and mining land were: agricultural land > construction land > ecological land. Therefore, under the condition of safe soil use, priority should be given to the agricultural land type.

## Supporting information

S1 Dataset (XLSX)
